# A study of triple-mass diffusion species and energy transfer in Carreau–Yasuda material influenced by activation energy and heat source

**DOI:** 10.1038/s41598-022-13890-y

**Published:** 2022-06-17

**Authors:** Muhammad Sohail, Umar Nazir, Essam R. El-Zahar, Hussam Alrabaiah, Poom Kumam, Abd Allah A. Mousa, Kanokwan Sitthithakerngkiet, Choonkil Park

**Affiliations:** 1 Department of Mathematics, Khwaja Fareed University of Engineering and Information Technology, Rahim Yar Khan, 64200 Pakistan; 2 Department of Applied Mathematics and Statistics, Institute of Space Technology, P.O. Box 2750, Islamabad, 44000 Pakistan; 3 Department of Mathematics, College of Science and Humanities in Al-Kharj, Prince Sattam Bin Abdulaziz University, P.O. Box 83, Al-Kharj, 11942 Saudi Arabia; 4 Department of Basic Engineering Science, Faculty of Engineering, Menoufia University, Shebin El-Kom, 32511 Egypt; 5 College of Engineering, Al Ain University, Al Ain, UAE; 6 Department of Mathematics, Tafila Technical University, Tafila, Jordan; 7 Center of Excellence in Theoretical and Computational Science (TaCS-CoE) and KMUTT Fixed Point Research Laboratory, Room SCL 802 Fixed Point Laboratory, Science Laboratory Building, Departments of Mathematics, Faculty of Science, King Mongkut’s University of Technology Thonburi (KMUTT), 126 Pracha-Uthit Road, Bang Mod, Thung Khru, Bangkok, 10140 Thailand; 8 Department of Medical Research, China Medical University Hospital, China Medical University, Taichung, 40402 Taiwan; 9 Department of Mathematics, College of Science, Taif University, P.O. Box 11099, Taif, 21944 Saudi Arabia; 10 Intelligent and Nonlinear Dynamic Innovations Research Center, Department of Mathematics, Faculty of Applied Science, King Mongkut’s University of Technology North Bangkok (KMUTNB), 1518, Wongsawang, Bangsue, Bangkok, 10800 Thailand; 11 Research Institute for Natural Sciences, Hanyang University, Seoul, 04763 Korea

**Keywords:** Mathematics and computing, Nanoscience and technology

## Abstract

The mechanism of thermal transport can be enhanced by mixing the nanoparticles in the base liquid. This research discusses the utilization of nanoparticles (tri-hybrid) mixture into Carreau–Yasuda material. The flow is assumed to be produced due to the stretching of vertical heated surface. The phenomena of thermal transport are modeled by considering Joule heating and heat generation or absorption involvement. Additionally, activation energy is engaged to enhance heat transfer rate. The mathematical model composing transport of momentum, heat and mass species is developed in Cartesian coordinate system under boundary layer investigation in the form of coupled nonlinear partial differential equations. The complex partial differential equations are converted into coupled nonlinear ordinary differential equations by using the appropriate similarity transformation. The conversion of PDEs into ODEs make the problem easy to handle and it overcome the difficulties to solve the PDEs. The transformed ordinary differential equations are solved with the help of help of finite element scheme. The obtained solution is plotted against numerous involved parameters and comparative study is established for the reliability of method and accuracy of obtained results. An enhancement in fluid temperature is recorded against magnetic parameter and Eckert number. Also, decline in velocity is recorded for Weissenberg number and concentration is controlled against higher values of Schmidt number. Furthermore, it is recommended that the finite element scheme can be implemented to handle complex coupled nonlinear differential equation arising in modeling of several phenomena occurs in mathematical physics.

## Introduction

Transport of heat in fluid flows has much importance due to their usage in many industrial applications. Researchers have great interest on these medium and mechanisms which are favorable for thermal transport. Carreau Yasuda liquid is known as non-Newtonian martial which is applicable in colloidal suspension, manufacturing, engineering problems and fermentation industry. Bhatti et al.^1^ investigated consequences of energy transfer into Intra-uterine particle motion using tapered heated channel. Bhatti and Abdelsalam^2^ studied behavior of Ree‐Eyring liquid under action of magnetic parameter considering by irreversible process. Bilal et al.^3^ discussed features of mathematical model containing microorganisms inserting approach of hybrid nanoparticles whereas flow has been induced by wavy fluctuating heated disk. Elmaboud and Abdelsalam^4^ captured model based on generalized Burger's liquid using effects of magnetic parameter in an annulus. Abumandour et al.^5^ studied peristaltic thrusting in the presence of nanoparticles trough vertical pipe including thermal-viscosity. Haq et al.^6^ captured consequences of energy transfer inserting SWCNTs in trapezoidal cavity solved by finite element method. The involvement of nanoparticles for the enhancement of heat transport in thermal exchanges under several important effects computational study was presented by Bondareva et al.^7^ to analyze the thermal transport by mixing different nanoparticles indifferent phase changing materials. Mallawi and ullah^8^ examined the continuation of slip in Darcy–Forchheimer inclined plane medium containing the mixture of hybrid nanoparticles. They derived the flow governing equation in the form of partial differential equations by engaging boundary layer survey and solved the resulting transformed ordinary differential equations by incorporating similarity transformation with the help of homotopic procedure. They monitored the decline in velocity field by augmenting the values of slip parameter. Algehyne et al.^9^ studied the inclusion of ternary hybrid nanoparticles mixture in pseudo-plastic material past over a heated porous surface in the presence of heat generation and modified heat flux (Cattaneo–Christov model). They analyzed the shear thinning and thickening behavior of considered model for different values of power law index. They engaged a powerful numerical scheme namely finite element procedure to handle the model equations. They found the description in fluid velocity against porosity parameter.

Modeling of fluid flows over a stretching surface got remarkable consideration due to their industrial wider applications. For instance, Dadheech et al.^10^ implemented magnetic field in energy transfer phenomena based on natural convention inserting approach of hybrid nanoparticles and nanofluid. They investigated comparative analysis among nanofluid and hybrid nanoparticles. Khan and Pop^11^ studied the stretched viscous nanofluid boundary value problem past over a linear stretching sheet. They have shown the increase in temperature against Brownian motion parameter and depreciation in concentration field. Moreover, validity of result is expressed by comparing the obtained solution as a limiting case of previously published findings. Rajagopal et al.^12^ presented the numerical study on second grade liquid past over a stretching sheet. Chabani et al.^13^ used hybrid nanoparticles to obtain maximum amount of energy transfer in triangular enclosure. Shafiq et al.^14^ developed model hyperbolic tangent liquid inserting role of bioconvective flow with nanoparticles over porous surface. Saeed et al.^15^ investigated thermal enhancement in couple stress liquid using hybrid nanoparticles approach in the presence of Darcy–Forchheimer theory. Saeed et al.^16^ studied physical significance of nanoparticles over a stretching surface via convective energy transfer. Ullah et al.^17^ used the Lie group similarity analysis to handle the problem of non-Newtonian model with thermal transport. They mentioned that higher values of magnetic retard the flow. Some important contributions are covered in^18–21^. Elkoumy et al.^22^ discussed influences of Maxwell liquid in heat energy in the presence of peristaltic flow under action of Hall current and magnetic field. Abdelsalam^23^ studied electro-magnetically containing swimming sperms in the presence self-propulsion in heated channel. Eldesoky et al.^24^ discussed thermal aspects in peristaltically induced incorporating slip conditions in catheterized heated pipe. Bhatti and Abdelsalam^25^ studied impacts of hybrid nanoparticles in peristaltic propulsion under action of magnetic effects. Marzougui et al.^26^ captured features of magnetic field in entropy generation and energy transfer in the presence of nanofluid. Pushpa et al.^27^ determined phenomena of convective flow inserting approach of nanofluid in thin baffle. Rasool et al.^28^ discussed model of Second grade liquid considering features of thermal radiation and viscous dissipation using Darcy–Forchheimer model. Shafiq et al.^29^ investigated mechanism magnetic field in the presence of Darcy–Forchheimer theory in nano-Casson material. Kumar et al.^30^ studied phenomena regarding enhancement of heat transfer in ferromagnetic flow in the occurrence of hybrid nanoparticles considering with solar radiation. Ganesh Kumar et al.^31^ performed modeling of heat transfer implementing nanoparticles considering various shapes effects over a moving heated frame numerically simulated by least square approach. Kumar et al.^32^ analyzed study of thermal energy enhancement due to carbon nanotubes in heated channel (convergent/divergent) under the occurrence of Darcy–Forchheimer medium. Souayeh et al.^33^ investigated characterizations of thermal radiation in energy transfer involving rheology of ferromagnetic in the presence of nanomaterial based on dusty fluid using slip conditions. Kumar et al.^34^ discussed features of Maxwell liquid in Double-diffusive free approach related to conservative heat transfer including thermal radiation over a heated surface. Kumar et al.^35^ studied double diffusion phenomena in convective flow including Casson liquid involving the concept of thermal radiation using slip conditions and thermal radiation. Kumar et al.^36^ discussed thermal features in viscoelastic fluid inserting nanofluid under action of thermal radiation implementing convective boundary conditions past a stretching heated surface. Ganesh Kumar^37^ developed energy transfer model in term of three dimensional flows in the presence of thermal radiation and nanoparticles over heated surface. Kumar et al.^38^ investigated impacts of non-Newtonian liquid considering nanofluid and slip factor across a heated surface. Ali et al.^39^ analyzed modeling of blood behavior in stenosis simulated by finite difference approach. Khan et al.^40^ modeled problem regarding energy transfer including anomalous diffusion amorphous semiconductors implementing fractional calculus. Hussain et al.^41^ studied mixed convective heat transfer in presence of CNTs nanofluid considering reactions and variable viscosity. Irfan et al.^42^ evaluated thermal aspects in term of convective heat transport using new mass flux approach in Carreau liquid. Ali et al.^43^ studied dynamics behavior regarding Josephson junction using fractional calculus procedure. Hussain et al.^44^ discussed features of thermal flux in Jeffery flow using concept of heat source past stretching surface. Hussain et al.^45^ studied thermal and solute effects in hybrid nanoparticles using chemical reaction over curved surface involving various shapes effects. Irfan et al.^46^ investigated theoretical analysis of Carreau liquid in heat and mass fluxes using new concept of activation energy under magnetic influence. Rafiq et al.^47^ studied thermal features of Maxwell liquid in the presence of thermal radiation including suspension of nanoparticles.

Available study shows that no one attempted the ternary hybrid nanoparticles mixed Carreau–Yasuda model numerically via finite element scheme along with activation energy. The utilization of viscous dissipation, Joule heating in the mixture of ternary hybrid nanoparticles is an important novel contribution to the existing body of knowledge. This report is organized as follows.

- Literature survey is reported in “Introduction” section;
- Modeling under several important physical aspects with similarity analysis is included in “Transportation of mass diffusion species” section;
- Utilized scheme is explained in “Numerical method for solution” section;
- Results are analyzed in “Results and discussion” section and important findings are reported in “Conclusions” section. Further, tri-hybrid nanoparticles are made by mixture of silicon dioxide, aluminum oxide and titanium oxide in ethylene glycol.

## Transportation of mass diffusion species

**Figure 1:**
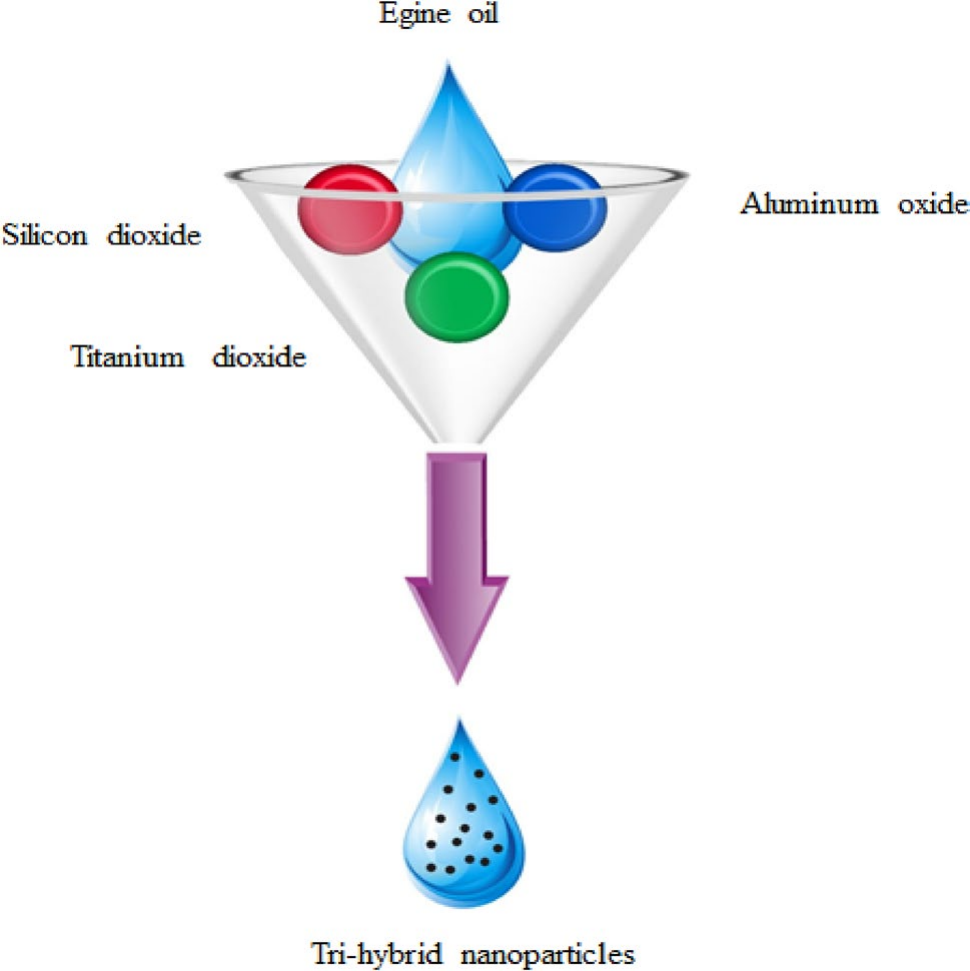
Development of tri-hybrid nanofluid.

**Figure 2:**
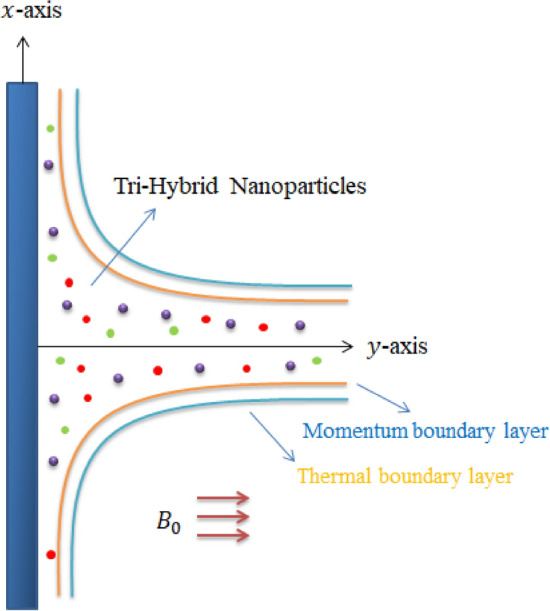
Model of 2D-streching surface.

An enhancement in motion of Tri-hybrid nanoparticles in Carreau Yasuda liquid over a vertical heated surface is observed which is shown in Fig. 1. Two dimensional flow, thermal energy and concentration is carried out. Bouncy force is appeared while variable magnetic field is taken out. It is noticed that magnetic field is assumed as a $\left[ {0, \frac{{B_{0} A}}{x}, 0} \right]$ to make dimensionless magnetic parameter while velocity regarding stretching surface is taken as $U_{w} = ax.$ Double diffusion species along with hybrid nanoparticles and tri-hybrid nanofluid is addressed. Motion of Tri-hybrid nanoparticles is occurred due to movement of walls along y-direction. Features related to viscous dissipation, heat generation and Joule heating are inserted in heat transfer phenomena. Transport of mass species is occurred in the presence of activation energy and chemical reaction. Following physical actions^48^ are considered below:

- The rheology of Carreau Yasuda liquid is observed;
- Triple species along with activation energy and chemical reaction is addressed;
- Heat generation phenomena is taken out;
- Effects related viscous dissipation and Joule heating are considered;
- Composite of $Al_{2} O_{3} , TiO_{2}$ and $MoS_{2}$ is called tri-hybrid nanoparticles whereas base fluid is considered as an engine oil;
- Figure 2 reveals mixture of Tri-hybrid nanoparticles. The development physical sequences in term mathematical model. Steady flow mode is considered two dimensional in the presence of Carreau Yasuda liquid. Transportation of triple mass diffusion and energy transfer involving impacts of chemical reaction, heat source, viscous dissipation and Joule heating. Approach of boundary layer approximations is used in conservation laws. The reduced form of PDEs are derived as

Physical actions are visulaized in form PDEs and non-linear PDEs^48,49^ are formulated as:

1 $$\frac{{\partial \check{u}}}{{\partial x}} + \frac{{\partial \check{v}}}{{\partial y}} = 0,$$

2 $$\begin{aligned} \check{u}\frac{{\partial \check{u}}}{{\partial x}} + {\text{ }}v\frac{{\partial \check{u}}}{{\partial y}} & = G\Upsilon _{{thnf}} \left( {T - T_{\infty } } \right) + G\beta _{{thnf}} \left( {C - C_{{a\infty }} } \right) + G\alpha _{{thnf}} \left( {C - C_{{b\infty }} } \right) \\ & \quad - \frac{{A^{2} \sigma _{{thnf}} B_{0}^{2} }}{{x^{2} \rho _{{thnf}} }}{\text{ }}\check{u} + \nu _{{thnf}} \frac{{\partial ^{2} \check{u}}}{{\partial y^{2} }} + \Lambda ^{d} \nu _{{thnf}} \left( {\frac{{m - 1}}{d}} \right)\left( {d + 1} \right)\frac{{\partial ^{2} \check{u}}}{{\partial y^{2} }}\left( {\frac{{\partial ^{2} \check{u}}}{{\partial y^{2} }}} \right)^{d} \\ \end{aligned}$$

3 $$\begin{aligned} & \check{u}\frac{{\partial T}}{{\partial x}} + {\text{ }}\check{v}\frac{{\partial T}}{{\partial y}} = \frac{{K_{{thnf}} }}{{\left( {\rho C_{p} } \right)_{{thnf}} }}\frac{{\partial ^{2} T}}{{\partial y^{2} }} + \frac{{\sigma _{{thnf}} B_{0}^{2} }}{{\left( {\rho C_{p} } \right)_{{thnf}} }}\check{u}^{2} + \frac{{\mu _{{thnf}} }}{{\left( {\rho C_{p} } \right)_{{thnf}} }}\left( {\frac{{\partial \check{u}}}{{\partial y}}} \right)^{2} \\ & \quad + \frac{{\mu _{{thnf}} }}{{\left( {\rho C_{p} } \right)_{{thnf}} }}\left( {\frac{{m - 1}}{d}} \right)\Lambda ^{d} \left( {\frac{{\partial \check{u}}}{{\partial y}}} \right)^{2} \left( {\frac{{\partial \check{u}}}{{\partial y}}} \right)^{d} + \frac{{Q\sigma _{{thnf}} }}{{\left( {\rho C_{p} } \right)_{{thnf}} }}\left( {T - T_{\infty } } \right), \\ \end{aligned}$$

4 $$\check{u}\frac{{\partial C_{a} }}{{\partial x}} + {\text{ }}\check{v}\frac{{\partial C_{a} }}{{\partial y}} = D_{{S_{a} }} \frac{{\partial ^{2} C_{a} }}{{\partial y^{2} }} - k_{1} \left( {C - C_{{a\infty }} } \right) - K_{r}^{2} \left( {C - C_{{a\infty }} } \right)\left( {\frac{T}{{T_{\infty } }}} \right)^{n} e^{{\frac{{ - E_{a} }}{{K_{0} }}}} ,$$

5 $$\check{u}\frac{{\partial C_{b} }}{{\partial x}} + {\text{ }}\check{v}\frac{{\partial C_{b} }}{{\partial y}} = D_{{S_{b} }} \frac{{\partial ^{2} C_{b} }}{{\partial y^{2} }}.$$

Desired transformations in the presence of hybrid nanoparticles are

6 $$\begin{aligned} & \check{u} = ax,\;\check{v} = V_{w} ,T = T_{w} ,C_{a} = C_{{aw}} ,C_{b} = C_{{bw}} :y = 0, \\ & \check{u} \to 0,T \to T_{\infty } ,C_{a} \to C_{{aw}} ,C_{b} \to C_{{b\infty }} :y \to \infty . \\ \end{aligned}$$

7 $$\theta \left( \xi \right) = \frac{{T - T_{\infty } }}{{T_{w} - T_{\infty } }},{\text{ }}\check{v} = - \left( {a\nu _{f} } \right)^{{1/2}} F,\;{\text{ }}\check{u} = axf^{\prime } ,\;\eta = y\left( {\frac{a}{{\nu _{f} }}} \right)^{{1/2}} ,\Phi \left( \xi \right) = \frac{{C_{a} - C_{{a\infty }} }}{{C_{{aw}} - T_{{a\infty }} }},\phi \left( \xi \right) = \frac{{C_{b} - C_{{b\infty }} }}{{C_{{bw}} - C_{{b\infty }} }}.$$

It is noticed that Eq. (7) is used to obtain system of ODEs in Eqs. (1)–(6) modeled ODEs with dimensionless BCs

8 $$F^{{\prime \prime \prime }} + \left( {\frac{{m - 1}}{d}} \right)W^{d} \left( {d + 1} \right)\left( {F^{{\prime \prime }} } \right)^{d} F^{{\prime \prime \prime }} + \frac{{\nu _{f} }}{{\nu _{{thnf}} }}\delta _{1} \theta + \frac{{\nu _{f} }}{{\nu _{{thnf}} }}\delta _{2} \phi + \frac{{\nu _{f} }}{{\nu _{{thnf}} }}\delta _{3} \Phi + \frac{{\nu _{f} }}{{\nu _{{thnf}} }}FF^{{\prime \prime }} - \frac{{\nu _{f} }}{{\nu _{{thnf}} }}F^{{\prime 2}} - \frac{{\sigma _{{thnf}} }}{{\sigma _{f} }}M^{2} F^{\prime } = 0,$$

9 $$\theta^{\prime\prime} - \frac{{k_{f} }}{{k_{thnf} }}\frac{{\left( {1 - \varphi_{1} } \right)^{ - 2.5} PrEcM^{2} }}{{\left( {1 - \varphi_{3} } \right)^{2.5} \left( {1 - \varphi_{2} } \right)^{2.5} }}\left( {1 + \frac{m - 1}{d}} \right)W^{d} \left( {F^{\prime\prime}} \right)^{d} F^{{\prime\prime}{2}} + \frac{{k_{f} }}{{k_{thnf} }}PrH_{t} \theta + \frac{{k_{f} }}{{k_{thnf} }}\frac{{Ec\left( {1 - \varphi_{3} } \right)^{ - 2.5} }}{{\left( {1 - \varphi_{1} } \right)^{2.5} \left( {1 - \varphi_{2} } \right)^{2.5} }}M^{2} PrF^{{\prime}{2}} + \frac{{k_{f} }}{{k_{thnf} }}\frac{{\left( {\rho c_{p} } \right)_{thnf} }}{{\left( {\rho c_{p} } \right)_{f} }}PrF\theta^{\prime} = 0,$$

10 $$\phi^{\prime\prime} + \frac{{\left( {1 - \varphi_{3} } \right)^{ - 2.5} Sc}}{{\left( {1 - \varphi_{2} } \right)^{2.5} \left( {1 - \varphi_{1} } \right)^{2.5} }}F\phi^{\prime} - \frac{{\left( {1 - \varphi_{3} } \right)^{ - 2.5} Sc}}{{\left( {1 - \varphi_{2} } \right)^{2.5} \left( {1 - \varphi_{1} } \right)^{2.5} }}\left( {1 + {\updelta }\theta } \right)^{n} \phi \sigma e^{{\left( {\frac{ - E}{{1 + {\updelta }\theta }}} \right)}} - K_{c} \frac{{\left( {1 - \varphi_{3} } \right)^{ - 2.5} Sc}}{{\left( {1 - \varphi_{2} } \right)^{2.5} \left( {1 - \varphi_{1} } \right)^{2.5} }}\phi = 0,$$

11 $$\Phi ^{{\prime \prime }} - \frac{{\left( {1 - \varphi _{3} } \right)^{{ - 2.5}} Le}}{{\left( {1 - \varphi _{2} } \right)^{{2.5}} \left( {1 - \varphi _{1} } \right)^{{2.5}} }}\left( {F\Phi ^{\prime } } \right) = 0.$$

Dimensionless BCs are

12 $$\begin{aligned} \theta \left( 0 \right) & = 1 = \phi \left( 0 \right) = \Phi \left( 0 \right),\;\theta \left( \infty \right) \to 0,F\left( 0 \right) = 0, \\ F^{\prime } \left( 0 \right) & = 1,\phi \left( \infty \right) \to 0,\;F\left( \infty \right) \to 0,\;\Phi \left( \infty \right) \to 0. \\ \end{aligned}$$

It is noticed that present developed problem is known as non-Newtonian model in the presence of Carreau Yasuda material. The present model can be reduced in Newtonian model by implanting $\varphi_{1} = W = \varphi_{2} = \varphi_{3} = 0$ in governing equations. The correlations among hybrid nanoparticles and nanoparticles are as under and their properties are listed in Table 1.

13 $$\rho_{Thnf} = \left( {1 - \varphi_{1} } \right)\left\{ {\left( {1 - \varphi_{2} } \right)\left[ {\left( {1 - \varphi_{3} } \right)\rho_{f} + \varphi_{3} \rho_{3} } \right] + \varphi_{2} \rho_{2} } \right\} + \varphi_{1} \rho_{1} ,$$

14 $$\frac{{\mu_{f} }}{{\left( {1 - \varphi_{3} } \right)^{2.5} \left( {1 - \varphi_{2} } \right)^{2.5} \left( {1 - \varphi_{1} } \right)^{2.5} }}, \frac{{K_{hnf} }}{{K_{nf} }} = \frac{{K_{2} + 2K_{nf} - 2\varphi_{1} \left( {K_{nf} - K_{2} } \right)}}{{K_{2} + 2K_{nf} + \varphi_{2} \left( {K_{nf} - K_{2} } \right)}},$$

15 $$\frac{{K_{Thnf} }}{{K_{hnf} }} = \frac{{K_{1} + 2K_{hnf} - 2\varphi_{1} \left( {K_{hnf} - K_{1} } \right)}}{{K_{1} + 2K_{hnf} + \varphi_{1} \left( {K_{hnf} - K_{1} } \right)}}, \frac{{K_{nf} }}{{K_{f} }} = \frac{{K_{3} + 2K_{f} - 2\varphi_{3} \left( {K_{f} - K_{3} } \right)}}{{K_{3} + 2K_{f} + \varphi_{3} \left( {K_{f} - K_{3} } \right)}},$$

16 $$\frac{{\sigma_{Tnf} }}{{\sigma_{hnf} }} = \frac{{\sigma_{1} \left( {1 + 2\varphi_{1} } \right) - \varphi_{hnf} \left( {1 - 2\varphi_{1} } \right)}}{{\sigma_{1} \left( {1 - \varphi_{1} } \right) + \sigma_{hnf} \left( {1 + \varphi_{1} } \right)}}, \frac{{\sigma_{hnf} }}{{\sigma_{nf} }} = \frac{{\sigma_{2} \left( {1 + 2\varphi_{2} } \right) + \varphi_{nf} \left( {1 - 2\varphi_{2} } \right)}}{{\sigma_{2} \left( {1 - \varphi_{2} } \right) + \sigma_{nf} \left( {1 + \varphi_{2} } \right)}},$$

17 $$\frac{{\sigma_{nf} }}{{\sigma_{f} }} = \frac{{\sigma_{3} \left( {1 + 2\varphi_{3} } \right) + \varphi_{f} \left( {1 - 2\varphi_{3} } \right)}}{{\sigma_{3} \left( {1 - \varphi_{3} } \right) + \sigma_{f} \left( {1 + \varphi_{3} } \right)}}.$$

**Table 1:** Thermal properties of hybrid nanoparticles with base fluid.

	$K$	$\sigma$	$\rho$
Engine oil	0.144	$0.125 \times 10^{ - 11}$	884
Aluminium oxide	32.9	$5.96 \times 10^{7}$	6310
Titanium dioxide	8.953	$2.4 \times 10^{6}$	4250
Silicon dioxide	1.4013	$3.5 \times 10^{6}$	2270

Equations (13)–(16) are represented correlations of tri-hybrid nanoparticles in base fluid whereas tri-hybrid nanofluid model is reduced into hybrid nanofluid model by implanting $\varphi_{3} = 0$ and nanofluid model is obtained using $\varphi_{3} = \varphi_{2} = 0$ in Eqs. (13)–(16). The properties of nanoparticles are shown in Table 1. It is mentioned that model related pure fluid is obtained considering $\varphi_{3} = \varphi_{2} = \varphi_{3} = 0.$ Physical involved parameters in dimensionless momentum equations are:

$$W = \left( {\frac{{a^{3} x^{2} 2{\Lambda }^{2} }}{{\nu_{f} }}} \right)^{1/2} , {\updelta } = \frac{{T_{w} - T_{\infty } }}{T},\delta_{1} = \frac{{\gamma GT_{0} }}{{2a\nu_{f} }}, \delta_{3} = \frac{{\alpha GC_{b0} }}{{2a\nu_{f} }}, M = \left( {\frac{{x^{2} A^{2} B_{0}^{2} \sigma_{f} }}{{a\rho_{f} }}} \right)^{1/2} ,$$

$$Pr = \frac{{\mu_{f} \left( {C_{p} } \right)_{f} }}{{k_{f} }}, Ec = \frac{{\left( {ax} \right)^{2} }}{{\left( {T_{w} - T_{\infty } } \right)\left( {C_{p} } \right)_{f} }}, H_{t} = \frac{Q}{{a\rho_{f} \left( {C_{p} } \right)_{f} }}, K_{c} = \frac{{k_{1} }}{a}, Sc = \frac{{D_{af} }}{{\nu_{f} }}, Le = \frac{{\nu_{f} }}{{D_{bf} }},$$

$$\delta_{2} = \frac{{\beta GC_{a0} }}{{2a\nu_{f} }}.$$

Drag force (skin friction coefficient) in view of Carreau Yasuda liquid under the action of hybrid nanoparticles are

18 $$\left( {Re} \right)^{\frac{1}{2}} C_{f} = \frac{{ - \left( {1 - \varphi_{3} } \right)^{ - 2.5} }}{{\left( {1 - \varphi_{1} } \right)^{2.5} \left( {1 - \varphi_{2} } \right)^{2.5} }}\left[ {1 + \frac{m - 1}{d}\left( {WF^{\prime \prime } \left( 0 \right)} \right)^{2} } \right]F^{\prime \prime } \left( 0 \right).$$

Rate of heat transfer in the presence of hybrid nanoparticles is

19 $$Nu = \frac{{xQ_{w} }}{{K_{f} \left( {T - T_{\infty } } \right)}}, Q_{w} = - K_{thnf} \frac{\partial T}{{\partial y}},$$

20 $$\left( {Re} \right)^{ - 1/2} Nu = \frac{{ - K_{thnf} }}{{K_{f} }}\theta^{\prime } \left( 0 \right).$$

Concentration gradient at wall of surface is

21 $$\left( {Re} \right)^{ - 1/2} Sh = \frac{{\left( {1 - \varphi_{3} } \right)^{ - 2.5} }}{{\left( {1 - \varphi_{1} } \right)^{2.5} \left( {1 - \varphi_{2} } \right)^{2.5} }}\varphi^{\prime } \left( 0 \right).$$

$Re = \frac{{xU_{w} }}{{\nu_{f} }}$ is known as Reynolds number.

## Numerical method for solution

**Figure 3:**
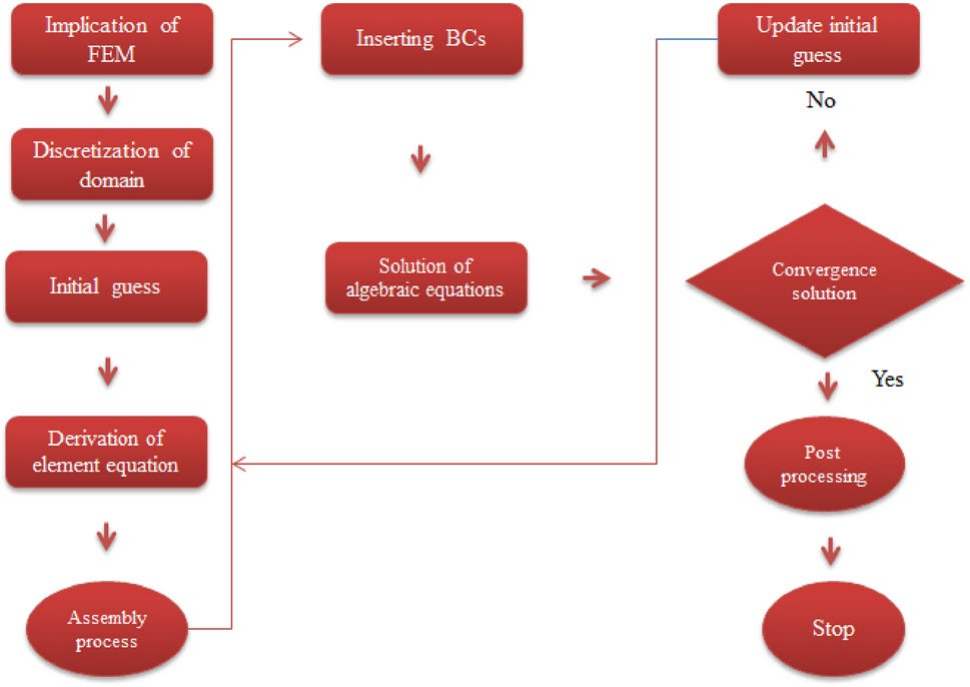
Flow chart related to FEM.

A system of ODEs within BCs is numerically solved using finite element approach. Discussion regarding finite element approach is addressed below. A finite element method is adopted to simulate numerical results of present model which is clearly explained via Fig. 3. A field related structural mechanics is used to develop finite element method. A phenomenal role of finite element method is that to tackle complex geometries, unstructured grids and curved cells with ease. An important advantage of FEM is that to divide problem into finite number of elements. A finite element method is observed as good method in view of accuracy analysis, convergence analysis and stability analysis rather than others numerical methods. The following advantages of implementing finite element method are listed below.

- Numerous of applications of finite element method are investigated in computational fluid mechanics problems;
- Complex types of geometries are tackled by FEM;
- Physical problems based on applied science are developed by FEM;
- It has ability to discretize the derivatives with very ease;
- An important role of FEM is that to solve various types of boundary conditions;
- FEM requires low investment and time rather than others numerical techniques.

- **Step-I**: Equations (6)–(7) within BCs are called strong form. It is noticed that collecting all terms of Eqs. (6)–(7) on one side and integrating it over each elements of domain are residuals. Such procedure is known as weighted (residual method) for the development of weak forms.
- **Step-II**: Weak form is developed using linear shape functions by implementing Galerkin finite element method. The residuals of present analysis are formulated as
  22 $$\int\limits_{{\eta _{e} }}^{{\eta _{{e + 1}} }} {w_{1} \left( {F^{\prime } - T} \right)d\xi = 0,}$$
  23 $$\mathop \smallint \limits_{{\eta _e}}^{{\eta _{e + 1}}} {w_2}\left[ {\begin{array}{*{20}{c}} {T'' + \left( {\frac{{m - 1}}{d}} \right){W^d}\left( {d + 1} \right){{\left( {T'} \right)}^d}{T^{''}} + \frac{{{\nu _f}}}{{{\nu _{thnf}}}}FT' - \frac{{{\nu _f}}}{{{\nu _{thnf}}}}{T^2}}\\ { - \frac{{{\sigma _{thnf}}}}{{{\sigma _f}}}{M^2}T + \frac{{{\nu _f}}}{{{\nu _{thnf}}}}{\delta _1}\theta + \frac{{{\nu _f}}}{{{\nu _{thnf}}}}{\delta _2}\phi + \frac{{{\nu _f}}}{{{\nu _{thnf}}}}{\delta _3}\Phi } \end{array}} \right]d\xi = 0,$$
  24 $$\int_{{\eta_{e} }}^{{\eta_{e + 1} }} {w_{3} } \left[ {\begin{array}{*{20}c} {\theta^{\prime\prime} - \frac{{k_{f} }}{{k_{thnf} }}\frac{{\left( {1 - \varphi_{1} } \right)^{ - 2.5} PrEcM^{2} }}{{\left( {1 - \varphi_{3} } \right)^{2.5} \left( {1 - \varphi_{2} } \right)^{2.5} }}\left( {1 + \frac{m - 1}{d}} \right)W^{d} \left( {T^{\prime}} \right)^{d} T^{{\prime}{2}} } \\ { + \frac{{k_{f} }}{{k_{thnf} }}\frac{{Ec\left( {1 - \varphi_{3} } \right)^{ - 2.5} }}{{\left( {1 - \varphi_{1} } \right)^{2.5} \left( {1 - \varphi_{2} } \right)^{2.5} }}M^{2} PrT^{2} + \frac{{k_{f} }}{{k_{thnf} }}\frac{{\left( {\rho c_{p} } \right)_{thnf} }}{{\left( {\rho c_{p} } \right)_{f} }}PrF\theta ^{\prime}} \\ { + \frac{{k_{f} }}{{k_{thnf} }}PrH_{t} \theta } \\ \end{array} } \right]d\xi = 0,$$
  25 $$\int\limits_{{\eta_{e} }}^{{\eta_{e + 1} }} w_{4} \left[ {\begin{array}{*{20}c} {\phi^{\prime\prime} + \frac{{\left( {1 - \varphi_{3} } \right)^{ - 2.5} Sc}}{{\left( {1 - \varphi_{2} } \right)^{2.5} \left( {1 - \varphi_{1} } \right)^{2.5} }}F\phi^{\prime} + K_{c} \frac{{\left( {1 - \varphi_{3} } \right)^{ - 2.5} Sc}}{{\left( {1 - \varphi_{2} } \right)^{2.5} \left( {1 - \varphi_{1} } \right)^{2.5} }}\phi } \\ { - \frac{{\left( {1 - \varphi_{3} } \right)^{ - 2.5} Sc}}{{\left( {1 - \varphi_{2} } \right)^{2.5} \left( {1 - \varphi_{1} } \right)^{2.5} }}\left( {1 + {\updelta }\theta } \right)^{n} \phi \sigma e^{{\left( {\frac{ - E}{{1 + {\updelta }\theta }}} \right)}} } \\ \end{array} } \right]d\xi = 0,$$
  26 $$\int\limits_{{\eta _{e} }}^{{\eta _{{e + 1}} }} {w_{5} } \left[ {\Phi ^{{\prime \prime }} - \frac{{\left( {1 - \varphi _{3} } \right)^{{ - 2.5}} Le}}{{\left( {1 - \varphi _{2} } \right)^{{2.5}} \left( {1 - \varphi _{1} } \right)^{{2.5}} }}\left( {F\Phi ^{\prime } } \right)} \right]d\xi = 0.$$
  The shape functions are developed as
  27 $$\psi_{j} = \left( { - 1} \right)^{j - 1} \left( {\frac{{ - \xi + \xi_{j - 1} }}{{ - \xi_{j} + \xi_{j + 1} }}} \right), i = 1, 2.$$
- **Step-III**: Assembly approach is utilized for the development of stiffness element whereas assembly approach is performed via assembly procedure of FEA. Stiffness elements are derived as
  28 $$K_{ij}^{14} = 0,K_{ij}^{11} = \int\limits_{{\eta_{e} }}^{{\eta_{e + 1} }} \left( {\frac{{d\psi_{j} }}{d\eta }\psi_{i} } \right)d\xi , K_{ij}^{12} = \int\limits_{{\eta_{e} }}^{{\eta_{e + 1} }} \left( {\psi_{j} \psi_{i} } \right)d\xi , B_{i}^{1} = 0,K_{ij}^{13} = 0,K_{ij}^{15} = 0,$$
  29 $$K_{ij}^{22} = \int\limits_{{\eta_{e} }}^{{\eta_{e + 1} }} \left[ {\begin{array}{*{20}c} { - \frac{{d\psi_{i} }}{d\eta }\frac{{d\psi_{j} }}{d\eta } - \left( {\frac{m - 1}{d}} \right)W^{d} \left( {d + 1} \right)\left( {\overline{{T^{\prime}}} } \right)^{d} \frac{{d\psi_{i} }}{d\eta }\frac{{d\psi_{j} }}{d\eta } + \frac{{\nu_{f} }}{{\nu_{thnf} }}\overline{F}\frac{{d\psi_{j} }}{d\eta }\psi_{i} } \\ { - \frac{{\nu_{f} }}{{\nu_{thnf} }}\overline{T}\psi_{j} \psi_{i} } \\ \end{array} } \right]d\xi ,$$
  30 $$K_{ij}^{23} = \int\limits_{{\eta_{e} }}^{{\eta_{e + 1} }} \left[ {\frac{{\nu_{f} }}{{\nu_{thnf} }}\delta_{1} \psi_{j} \psi_{i} } \right]d\xi ,K_{ij}^{24} = \int\limits_{{\eta_{e} }}^{{\eta_{e + 1} }} \left[ {\frac{{\nu_{f} }}{{\nu_{thnf} }}\delta_{2} \psi_{j} \psi_{i} } \right]d\xi ,K_{ij}^{35} = 0, K_{ij}^{43} = 0,$$
  31 $$K_{ij}^{25} = \int\limits_{{\eta_{e} }}^{{\eta_{e + 1} }} \left[ {\frac{{\nu_{f} }}{{\nu_{thnf} }}\delta_{3} \psi_{j} \psi_{i} } \right]d\xi , K_{ij}^{21} = 0, K_{ij}^{21} = 0, B_{i}^{2} = 0,K_{ij}^{24} = 0,B_{i}^{3} = B_{i}^{4} = 0,$$
  32 $$K_{ij}^{33} = \int\limits_{{\eta_{e} }}^{{\eta_{e + 1} }} \left[ { - \frac{{d\psi_{i} }}{d\eta }\frac{{d\psi_{j} }}{d\eta } + \frac{{k_{f} }}{{k_{thnf} }}\frac{{\left( {\rho c_{p} } \right)_{thnf} }}{{\left( {\rho c_{p} } \right)_{f} }}Pr\overline{F}\psi_{i} \frac{{d\psi_{j} }}{d\eta } + \frac{{k_{f} }}{{k_{thnf} }}PrH_{t} \psi_{j} \psi_{i} } \right]d\xi ,$$
  33 $$K_{ij}^{32} = \int\limits_{{\eta_{e} }}^{{\eta_{e + 1} }} \left[ {\begin{array}{*{20}c} { - \frac{{k_{f} }}{{k_{thnf} }}\frac{{\left( {1 - \varphi_{1} } \right)^{ - 2.5} PrEcM^{2} }}{{\left( {1 - \varphi_{3} } \right)^{2.5} \left( {1 - \varphi_{2} } \right)^{2.5} }}\left( {1 + \frac{m - 1}{d}} \right)W^{d} \left( {\overline{T^{\prime}} } \right)^{d} \overline{T^{\prime}} \psi_{i} \frac{{d\psi_{j} }}{d\eta }} \\ {\frac{{k_{f} }}{{k_{thnf} }}\frac{{PrEc\left( {1 - \varphi_{3} } \right)^{ - 2.5} }}{{\left( {1 - \varphi_{1} } \right)^{2.5} \left( {1 - \varphi_{2} } \right)^{2.5} }}M^{2} Pr\overline{T}\psi_{j} \psi_{i} } \\ \end{array} } \right]d\xi ,$$
  34 $$K_{ij}^{44} = \int\limits_{{\eta_{e} }}^{{\eta_{e + 1} }} \left[ {\begin{array}{*{20}c} { - \frac{{d\psi_{i} }}{d\eta }\frac{{d\psi_{j} }}{d\eta } + \frac{{\left( {1 - \varphi_{3} } \right)^{ - 2.5} Sc}}{{\left( {1 - \varphi_{2} } \right)^{2.5} \left( {1 - \varphi_{1} } \right)^{2.5} }}\overline{F}\psi_{i} \frac{{d\psi_{j} }}{d\eta }} \\ { + K_{c} \frac{{\left( {1 - \varphi_{3} } \right)^{ - 2.5} Sc}}{{\left( {1 - \varphi_{2} } \right)^{2.5} \left( {1 - \varphi_{1} } \right)^{2.5} }}\psi_{j} \psi_{i} } \\ { + - \frac{{\left( {1 - \varphi_{3} } \right)^{ - 2.5} Sc}}{{\left( {1 - \varphi_{2} } \right)^{2.5} \left( {1 - \varphi_{1} } \right)^{2.5} }}\left( {1 + {\updelta }\overline{\theta }} \right)^{n} \sigma e^{{\left( {\frac{ - \nu }{{1 + {\updelta }\overline{\theta }}}} \right)}} \psi_{j} \psi_{i} } \\ \end{array} } \right]d\xi , K_{ij}^{41} = 0,$$
  35 $$K_{ij}^{55} = \int\limits_{{\eta_{e} }}^{{\eta_{e + 1} }} \left[ { - \frac{{d\psi_{i} }}{d\eta }\frac{{d\psi_{j} }}{d\eta } - \frac{{\left( {1 - \varphi_{3} } \right)^{ - 2.5} Le}}{{\left( {1 - \varphi_{2} } \right)^{2.5} \left( {1 - \varphi_{1} } \right)^{2.5} }}\left( {\overline{F}\psi_{i} } \right)\frac{{d\psi_{j} }}{d\eta }} \right]d\xi , K_{ij}^{42} = 0.$$
- **Step-IV**: Picard linearization approach provides transformed algebraic system (linear equations).
- **Step-V**: Finally, system of linear algebraic equations is numerically solved within computational tolerance ($10^{ - 5}$). The stopping condition is listed below.
  36 $$\left| {\frac{{\delta_{i + 1} - \delta_{i} }}{{\delta^{i} }}} \right| < 10^{ - 5} .$$
- **Step-VI**: Table 2 reveals investigation of mesh-free;

**Table 2:** Simulations regarding mesh free investigations via 300 elements.

Number of elements	$F^{\prime}\left( {\frac{{\xi_{max} }}{2}} \right)$	$\theta \left( {\frac{{\xi_{max} }}{2}} \right)$	$\phi \left( {\frac{{\xi_{max} }}{2}} \right)$	$\varphi \left( {\frac{{\xi_{max} }}{2}} \right)$
30	0.5515980408	0.5514267410	0.5149044441	0.4936033240
60	0.5253982978	0.5252814384	0.7406090811	0.4723021221
90	0.5168385792	0.5167238550	0.4937938095	0.4752159807
120	0.5125909054	0.5124762908	0.4911292118	0.4619762026
150	0.5100545434	0.5099398431	0.4895277265	0.4573310032
180	0.5100443431	0.3312308320	0.4871245361	0.4542291236
210	0.4120134643	0.3302207021	0.4750125240	0.4530081633
240	0.4139033612	0.3290202121	0.4638104243	0.4532101332
270	0.4128214241	0.3292221312	0.4636213012	0.4530903206
300	0.4128031543	0.3292307121	0.4635302140	0.4530944237
- **Step-VII**: 300 elements are required to obtain convergence analysis. It is mentioned that programming of code is developed on Maple 18. A Maple 18 is implemented to design code related to finite element method. The validation of code is tested with already published works. After tested of code, simulations are done in view of physical situation against various parameters.

### Error analysis

Numerical values of Sherwood number, skin friction coefficients and Nusselt number are computed against various parameters using indigenous software (Maple 18) which simulate problem in iteration manner. It is mentioned that exact solution of developed model is not available. The stopping criterion is defined in Eq. (35). Therefore, Numerical values of Sherwood number, skin friction coefficients and Nusselt number are noticed in Table 5 when above stopping criterion is satisfied. Further, error analysis is simulated in Table 5.

37 $$Error = \left| {S^{N + 1} - S^{N} } \right|$$

where $N$ is used for number iterations and $S$ is called computational nodal value.

### Validation of results

It is noticed that comparative numerical values of Nusselt number is validated with published study^49^ neglecting impacts of tri-hybrid nanoparticle, viscous dissipation, Weissenberg number and heat generation number. Good agreements are found among present work and published work^49^ whereas comparative study is carried out by Table 3. Figure 3 reveals flow chart of FEA. Table 4 is tabulated validation regarding numerical results for Nusselt number with published study^48^.

**Table 3:** Validation among present work and published by^49^ in term of $- \left( {Re} \right)^{ - 1/2} Nu$ considering $\varphi_{1} , \varphi_{2} , \varphi_{3} , Ec, W, H_{t} = 0.$

$Pr$	Present work	Abolbashari et al.^49^
0.72	0.8088021240	0.80863135
1.00	1.0000201000	1.00000000
3.00	1.9230091201	1.92368259
10.0	3.7203304229	3.72067390

**Table 4:** Comparison of numerical study regarding $- \theta \left( 0 \right)$ with published work^48^ when $Ec = 0, H_{t} = 0, m = 0, \varphi_{1} = 0 \varphi_{2} = 0 \varphi_{3} = 0.$

$Pr$	Nawaz and Awais^48^	Present study
0.72	0.74454088	1.74401906
1.0	0.91192959	0.91197393
3.0	1.81548127	1.81568259

## Results and discussion

Aspects related to triple-diffusion species and energy transfer are studied towards a stretching heated sheet. Rheology of Carreau Yasuda material is inserted into fluid particles along with base fluid called engine oil inserted hybrid nanoparticles under an influence of magnetic field. Thermal aspects are based on heat generation, viscous dissipation and Joule heating phenomena whereas concentration aspects are considered as activation energy and chemical species. Such complex developed model is numerical simulated using finite element method. It noticed that ranges of parameters $0.1 \le W \le 3, {\updelta },{ }0.2 \le \delta_{3} \le 3, 0.1 \le \delta_{1} \le 3, 0.001 \le M \ge 1.5, 201 \le Pr \le 208, 0.3 \le Ec \le 5, - 2.0 \le H_{t} \le 5, - 1.5 \le K_{c} \le 0.3 \le , 0.4 \le Sc \le 6, 0.1 \le Le \le 3$ and $0.2 \le \delta_{2} \le 3$ are considered in simulations^50^. A detail and comprehensive discussion for concentration, flow behavior and heat energy is discussed below.

## Flow analysis and distribution in various parameters

**Figure 4:**
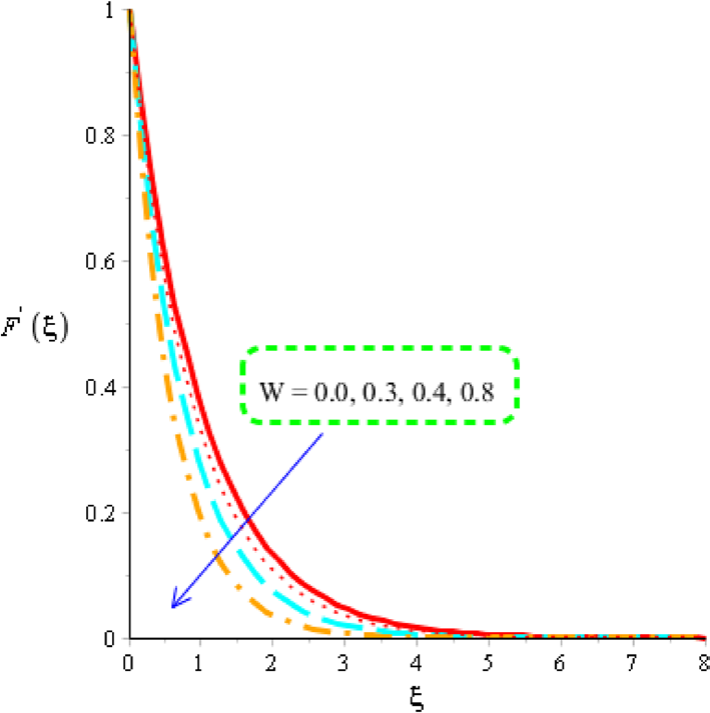
Distribution in velocity field versus $W.$

**Figure 5:**
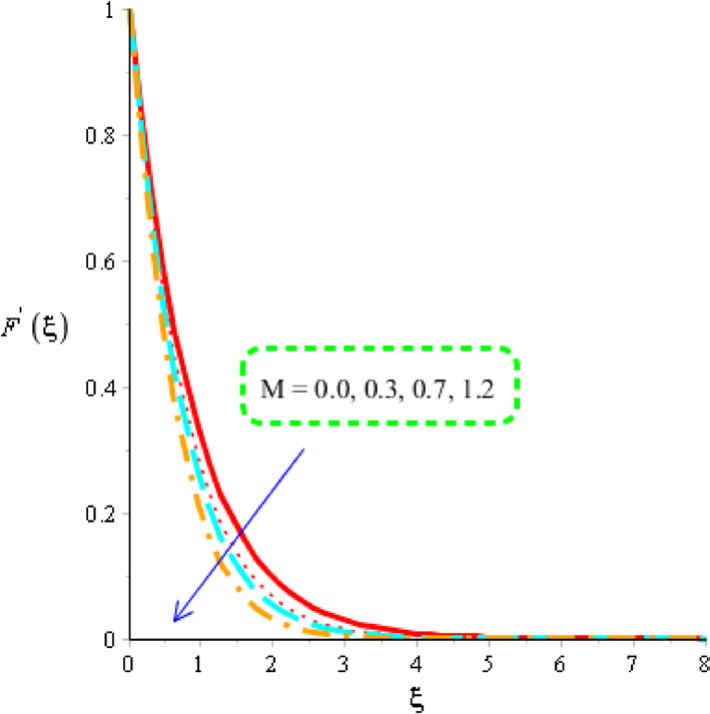
Distribution in velocity field versus $M.$

**Figure 6:**
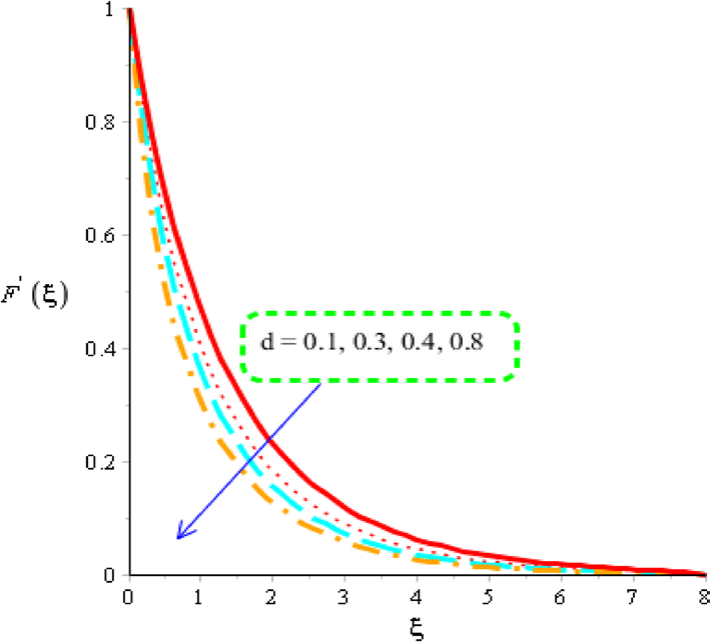
Distribution in velocity field versus $d.$

Flow of tri-hybrid nanoparticles is measured against variation in magnetic field number, Weissenberg number and fluid number whereas these influences are noticed by Figs. 4, 5 and 6. An influence of Weissenberg number on velocity field is addressed by Fig. 4. Velocity curves are decreasing function against an impact of $W.$ This decreasing impact of velocity curves is occurred due to using the concept of Weissenberg number. The termed related to Weissenberg number is defined as relation among viscous force and elastic force. An increase in Weissenberg number results an increment in viscous force. So, higher viscous force makes fluid layers more thick. It is noticed that fluid layers are decreased using higher numerical values of Weissenberg parameter. The measurement of motion into base fluid along with Carreau Yasuda liquid against magnetic field is addressed by Fig. 5. The reduction into motion particles is visualized when magnetic field is enhanced. This decreasing influence is happened due to Lorentz force. From mathematical point of view, Lorentz force is appeared as a negative force which is opposite against flow of nanoparticles. It is revealed that flow speed into motion is gradually reduced when magnetic field is gradually increased. Moreover, magnetic field is placed towards opposite flow regarding nanoparticles. Therefore, fluid becomes more viscous when magnetic field is applied. Momentum layers are based on numerical values of magnetic parameter. It estimated that velocity into fluidic particles is decreased when magnetic parameter is increased. Physically, occurrence of magnetic parameter is appeared using an impact of Lorentz force. Further, Lorentz force is force which is applied by magnetic field into fluidic particles. Lorentz force in momentum equation is appareled as negative force which produces significant friction in fluidic particles. Therefore, velocity of fluid is declined when magnetic number is increased. Thickness associated with momentum layers are also declined versus variation in magnetic parameter. An impact of $d$ is investigated into motion regarding particles considered by Fig. 6. The numerical values of $d$ are used to characterize fluids category. Decreasing role of velocity is noticed against higher values of $d.$ Such, kind effect is investigated by Fig. 6 including an impact of tri-hybrid nanoparticles. Here, the parameter is related to $\left( d \right)$ is known as fluid number. Physically, it is formulated due to presence of Carreau Yasuda liquid. Fluid is became more viscous against higher impact of $\left( d \right).$ A resistance force is produced into fluid particles when $\left( d \right)$ is increased.

### Heat energy analysis distribution in various parameters

**Figure 7:**
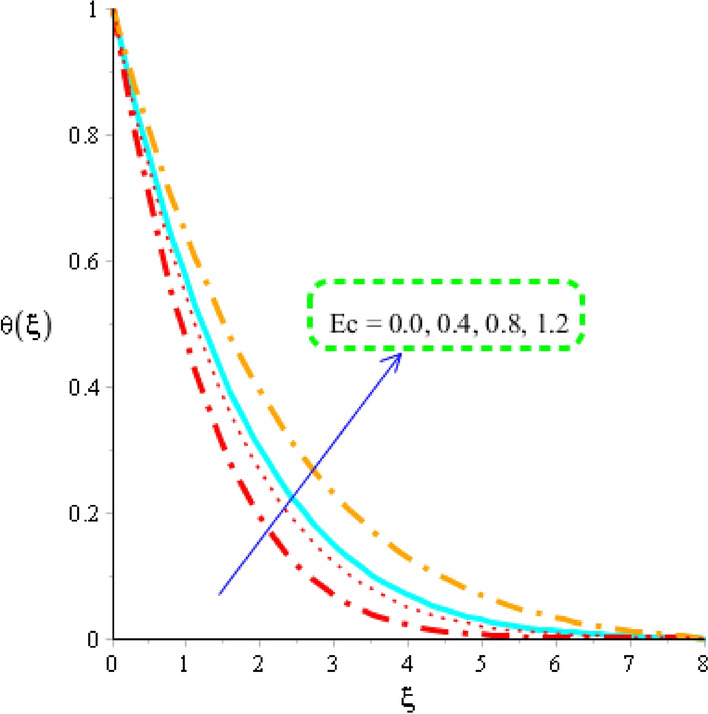
Distribution in temperature field versus $Ec.$

**Figure 8:**
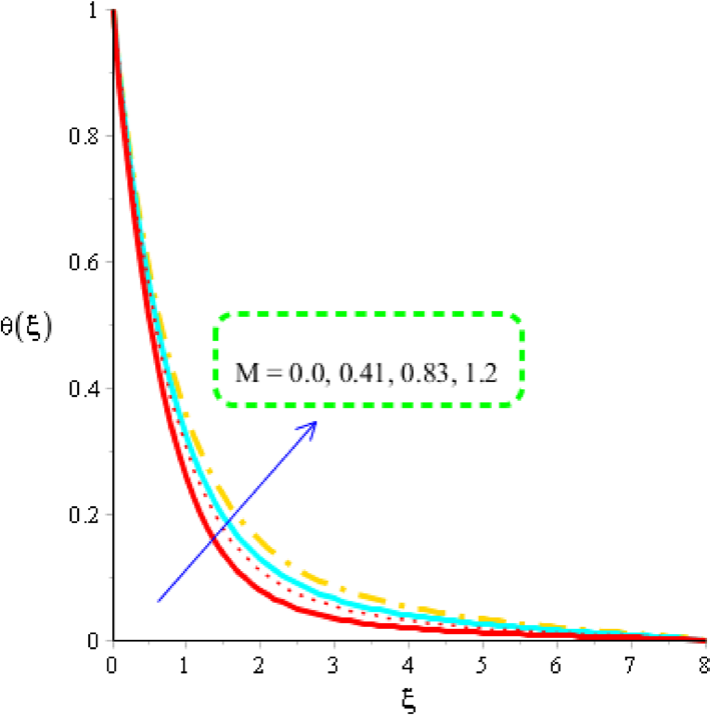
Distribution in temperature field versus $M.$

**Figure 9:**
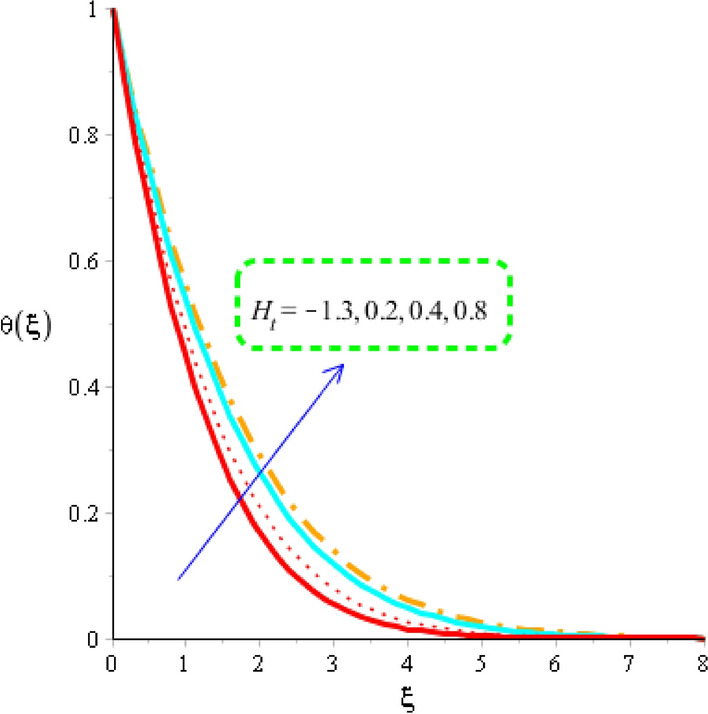
Distribution in temperature field versus $H_{t}$.

Figures 7, 8 and 9 reveal role of heat energy against Eckert number, heat generation number and magnetic field including base fluid called engine oil along with Carreau Yasuda martial. Figure 7 reveals an influence of Eckert number on an impact of heat energy. From Fig. 7, temperature into fluid particles is increased using higher values of Eckert number. From physical view, Eckert number means viscous dissipation of fluid particles. The direct relation among viscous dissipation and Eckert number is noticed. So, an increment in viscous dissipation reveals rate of work done of fluid particles is increased. Hence, heat energy is increased against higher values of Eckert number. Moreover, Eckert number is dimensionless number. Physically, it is defined as division among kinetic energy and enthalpy. So, an increment in Eckert number results higher kinetic energy of fluid particles. It is also used to measure the effect of self-heating phenomena into fluid particles. Thermal layers for the case of $Ec = 0$ is less than as compared for the case of $Ec \ne 0$. A role of magnetic parameter against temperature profile is measured by Fig. 8. It is mentioned that an impact of magnetic number is appeared because of Joule heating phenomenon. Moreover, Joule heating means conversion of electric current into heat energy. So, fast process regarding conversion of electric current into heat energy is occurred which is based on Joule heating procedure. Hence, useful effect of magnetic parameter is visualized on temperature profile considering tri-hybrid nanomaterial. The term related to Joule heating is depicted in dimensionless energy equation. It is dimensionless number which is used to measure fluidic temperature and amount of thermal energy. Thermal layers are based on variation of Joule heating term. It is found that thermal layers have increasing function versus magnetic field.

Figure 9 illustrates variation of heat energy versus applying numerical values of heat generation number whereas dual character of heat generation number is noticed. The dual behavior of $H_{t}$ is based on negative as well as positive values of heat generation number while negative values of $H_{t}$ is known as heat absorption and positive values of $H_{t}$ is termed as heat generation. Temperature profile is increased when heat generation number is increased. Physically, this increasing behavior of temperature profile is happened because of external heat source placed at wall.

### Mass species analysis and distribution in various parameters

**Figure 10:**
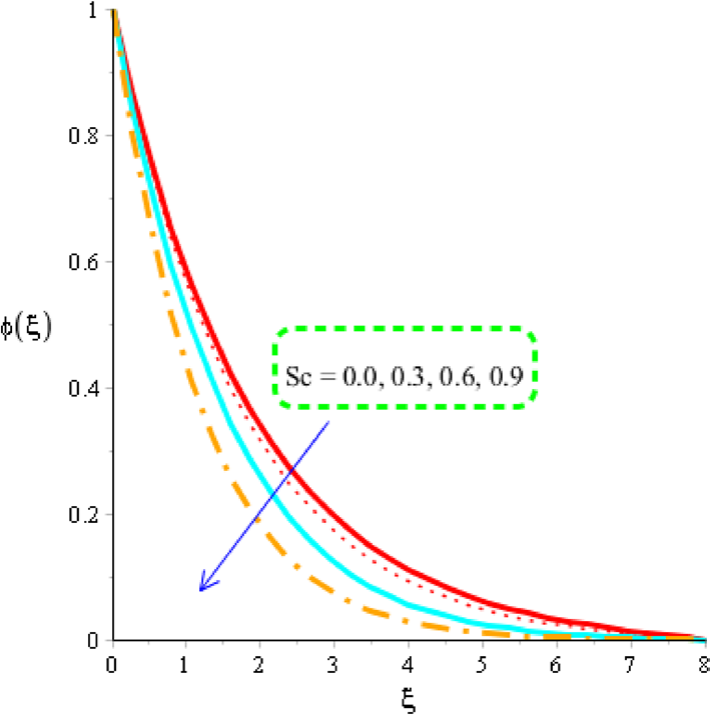
Distribution in $\phi \left( \eta \right)$ versus $Sc.$

**Figure 11:**
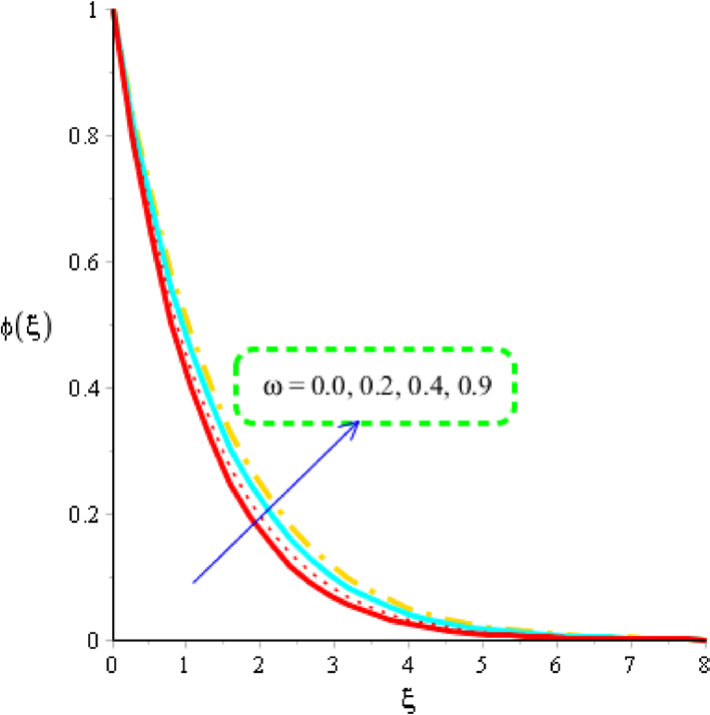
Distribution in $\phi \left( \xi \right)$ versus $\omega .$

**Figure 12:**
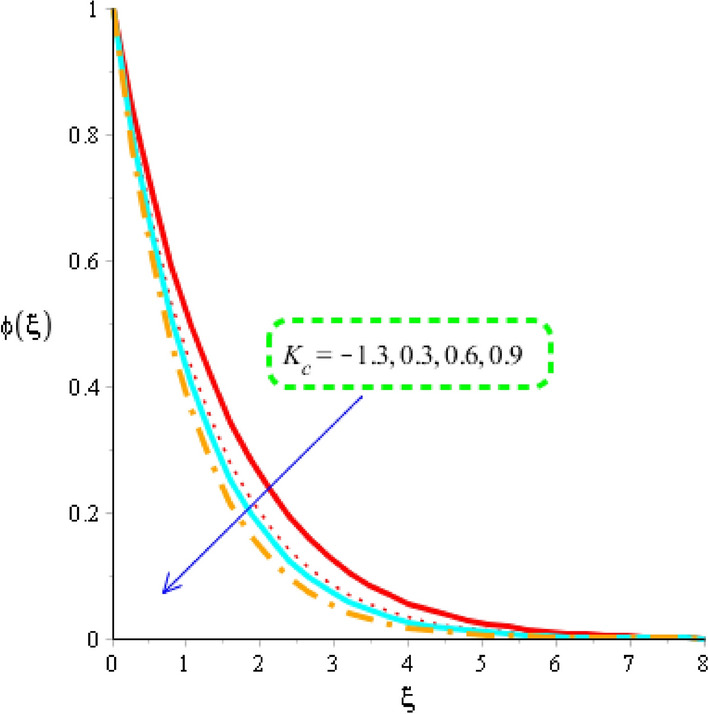
Distribution in $\phi \left( \xi \right)$ versus $K_{c} .$

**Figure 13:**
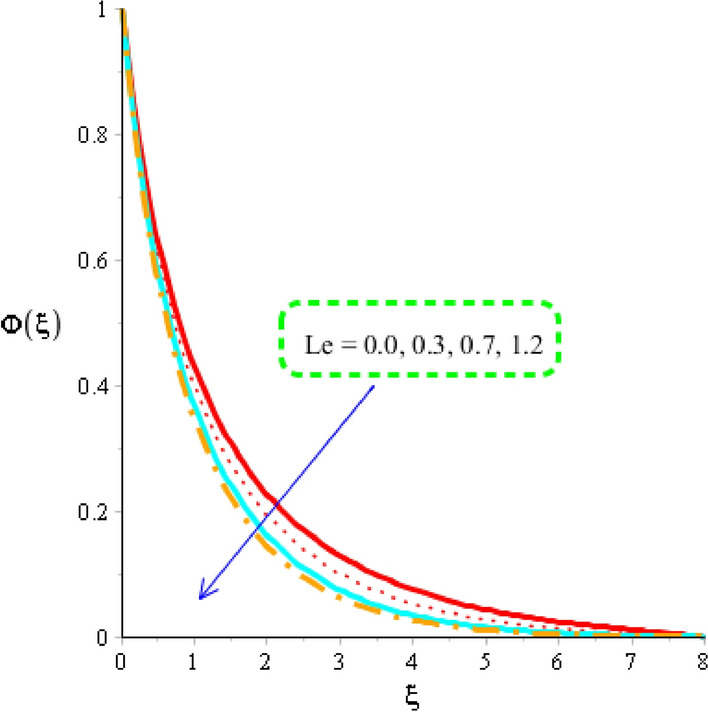
Distribution in ${\Phi }\left( \xi \right)$ versus $Le.$

The estimation of mass diffusion is investigated versus an influence of Schmidt number, activation number, chemical reaction number and Lewis parameter considered by Figs. 10, 11 and 12. Figure 10 is plotted to measure investigation among Schmidt number and diffusion of mass species. Diffusion into mass species becomes slow down versus an impact of Schmidt number. The Schmidt parameter is noticed as a division among mass diffusivity and momentum diffusivity. An inversely proportional relation is noticed in view Schmidt number versus mass diffusivity. Therefore, an increment into diffusion via mass species is occurred when Schmidt parameter is increased. The role of activation energy is observed on mass species conducted by Fig. 11. A significant impact of activation energy is investigated on mass species. It is noticed that procedure of diffusion of species becomes fast using higher values of activation of energy. Figure 12 predicts investigation of chemical reaction on mass species. From graphical point of view, concentration field is decreased versus higher impact of chemical reaction number. A decreasing impact of concentration profile is noticed when Lewis number is also increased is shown through Fig. 13.

### Analysis related to Nusselt number, drag force coefficient and Sherwood number

Table 5 is prepared for measure the influences against variation in Weissenberg number, magnetic number, heat generation number and chemical reaction number. It is noticed that argumentation is observed into motion via fluid particles versus the variation in Weissenberg number and magnetic parameter. Rate of heat transfer phenomena is decreased when magnetic parameter and Weissenberg number is enhanced. Role of heat generation is visualized as a significant for case of higher impact of heat generation number in view of fluid motion and thermal energy as well as for case of mass diffusion. An inclination into heat energy and mass species are captured against the variation in chemical reaction number in base fluid along with Carreau Yasuda liquid (Table 5).

**Table 5:** Numerical analysis of Nusselt number, skin friction coefficients, and Sherwood number with error analysis against variation in $W, M, H_{t}$ and $K_{c}$.

	$- \left( {Re} \right)^{\frac{1}{2}} C_{f}$	Error	$- \left( {Re} \right)^{ - 1/2} Nu$	Error	$- \left( {Re} \right)^{ - 1/2} Sh$	Error
$W\left( { = 0.0} \right)$	0.5007241803	$5 \times 10^{ - 9}$	0.07671888860	$1 \times 10^{ - 9}$	0.4497235394	$4 \times 10^{ - 9}$
$W\left( { = 0.4} \right)$	0.5006006674	$4 \times 10^{ - 9}$	0.07881268732	$2 \times 10^{ - 9}$	0.4497024213	$4 \times 10^{ - 9}$
$W\left( { = 0.8} \right)$	0.5004696036	$5 \times 10^{ - 9}$	0.08094075355	$9 \times 10^{ - 9}$	0.4498745213	$4 \times 10^{ - 9}$
$M\left( { = 0.0} \right)$	0.1429352440	$1 \times 10^{ - 9}$	0.1429352440	$1 \times 10^{ - 9}$	0.1283767519	$1 \times 10^{ - 9}$
$M\left( { = 0.4} \right)$	0.1510585728	$1.2 \times 10^{ - 9}$	0.37987164217	$3 \times 10^{ - 9}$	0.1766912266	$1.4 \times 10^{ - 9}$
$M\left( { = 0.8} \right)$	0.1704315997	$1 \times 10^{ - 9}$	0.57987164217	$5 \times 10^{ - 9}$	0.1961281131	$1 \times 10^{ - 9}$
$H_{t} \left( { = - 0.0} \right)$	0.1404308531	$1.3 \times 10^{ - 9}$	0.08539212196	$1 \times 10^{ - 9}$	0.1261274426	$1 \times 10^{ - 9}$
$H_{t} \left( { = 0.5} \right)$	0.1204302348	$1 \times 10^{ - 9}$	0.06780722846	$1 \times 10^{ - 9}$	0.1061268872	$1.1 \times 10^{ - 9}$
$H_{t} \left( { = 0.8} \right)$	0.1104292853	$1.7 \times 10^{ - 9}$	0.03035908385	$1.3 \times 10^{ - 9}$	0.0261260345	$3 \times 10^{ - 9}$
$K_{c} \left( { = 1.5} \right)$	0.1403862313	$1 \times 10^{ - 9}$	0.07049281878	$7 \times 10^{ - 9}$	0.1260873656	$1 \times 10^{ - 9}$
$K_{c} \left( { = 0.0} \right)$	0.1404825609	$1 \times 10^{ - 9}$	0.4067474293	$4 \times 10^{ - 9}$	0.1461738837	$1.2 \times 10^{ - 9}$
$K_{c} \left( { = 0.0} \right)$	0.1408623983	$1 \times 10^{ - 9}$	0.8197441934	$8 \times 10^{ - 9}$	0.1765150332	$1 \times 10^{ - 9}$

## Conclusions

Developed model including double diffusion effect over a moveable vertical surface is considered. A rheology related to Carreau Yasuda martial is analyzed along with Darcy’s Forchheimer model is addressed. A finite element scheme is utilized to simulate required results. The desired consequences are captured below.

- Convergence of problem is investigated carried by 300 elements;
- Higher viscosity is generated versus higher numerical values of magnetic number, fluid parameter and Weissenberg number;
- Maximum production in view of heat energy is obtained using argument impacts of magnetic parameter, Eckert number and heat generation number;
- Diffusion into fluid particles is slow down versus higher values of Schmidt number, Lewis number and chemical reaction number are increased;
- Ternary hybrid nanoparticles approach is visualized most significant to boost heat energy into fluid particles rather than hybrid nanoparticles approach;
- Coolant in automobiles and dynamics of fuel are applicable involvement of ternary hybrid nanoparticles;
- Remarkable achievement in heat transfer process which is applicable in industrial technologies, hybrid powered engines, fuel cells, microelectronics and industrial technologies;
- Developing model is applicable in printing process, electronic devices, temperature measurements, engineering process and food making, coolant in automobiles, fluidic dynamic process and solar systems.

## Data Availability

All the data is given within the manuscript.

## List of symbols

$$y,x$$: Space coordinates $$\left( {\text{m}} \right)$$
$$G$$: Gravitational acceleration $$\left( {\text{N}} \right)$$
$B_{0}$: Strength of magnetic field (T)
$thnf$: Ternary hybrid nanoparticles
$$\nu$$: Kinematic viscosity ($${\text{m}}^{2} \;{\text{s}}^{ - 1}$$)
$m$: Power law number
$$T$$: Fluid temperature ($${\text{K}}$$)
$$C_{p}$$: Specific heat ($${\text{JKg}}^{ - 1} \;{\text{K}}^{ - 1}$$)
$$\sigma$$: Electrical conductivity ($${\text{Wm}}^{ - 1}$$)
$$T_{\infty }$$: Ambient fluid ($${\text{K}}$$)
$$D_{ca} , D_{cb}$$: Mass diffusions ($${\text{m}}^{2} \;{\text{s}}^{ - 1}$$)
$$C_{a\infty } , C_{b\infty }$$: Ambient concentrations ($${\text{m}}^{2} \;{\text{s}}^{ - 1}$$)
$$T_{w}$$: Wall temperature ($${\text{K}}$$)
$$E_{a}$$: Activation parameter
$$\xi$$: Independent number
$a$: Stretching number along x-axis (${\text{s}}^{ - 1}$)
$${\Phi }, \varphi$$: Dimensionless concentrations
PDEs: Partial differential equations
$$\delta_{1} , \delta_{2} , \delta_{3}$$: Bouncy numbers
$$Pr$$: Prandtl number
$H_{t}$: Heat generation number
$$\varphi_{1, } \varphi_{3} , \varphi_{2}$$: Volume fractions
$$Le$$: Lewis number
$$U_{w}$$: Wall velocity ($${\text{ms}}^{ - 1}$$)
$$Nu$$: Nusselt number
$$Q_{w}$$: Wall flux
$n$: Fitted rate constant
$$w_{1} ,w_{4} , w_{3} , w_{2}$$: Weight functions
$$\widetilde{u }, \tilde{v}$$: Velocity components $$\left( {{\text{ms}}^{ - 1} } \right)$$
$$\beta_{1} , \beta_{2} , \beta_{3}$$: Bouncy parameters
$$\rho$$: Fluid density ($${\text{Kg}}\;{\text{m}}^{ - 3}$$)
$f, bf$: Fluid and base fluid
${\Lambda }$: Carreau Yasuda number
$d$: Carreau Yasuda number
$$K$$: Thermal conductivity ($${\text{Wm}}^{ - 1}$$)
$$\mu$$: Dynamic viscosity ($${\text{Kg}}\;{\text{m}}^{ - 1} \;{\text{s}}^{ - 1}$$)
$Q$: Heat source number
$$C_{a} , C_{b}$$: Concentrations ($${\text{Kg}}\;{\text{m}}^{ - 3}$$)
$$K_{1}$$: Chemical number ($${\text{s}}^{ - 1}$$)
FEM: Finite element method
$K_{r}$: Chemical reaction rate
$$\infty$$: Infinity
$$\theta$$: Dimensionless temperature
$$F$$: Dimensionless velocity
ODEs: Ordinary differential equations
$$W$$: Weisseneberg number
$M$: Magnetic field number
$$Ec$$: Eckert number
$$Sc$$: Schmidt number
$\delta$: Temperature difference number
$K_{c}$: Chemical reaction parameter
$$\nu$$: Kinematic viscosity ($${\text{m}}^{2} \;{\text{s}}^{ - 1}$$)
$$Sh$$: Sherwood number
FEA: Finite element approach
BCs: Boundary conditions
$$\psi_{j} , \psi_{i}$$: Shape functions
